# 3-Phenyl­pyridinium tetra­chlorido­aurate(III)

**DOI:** 10.1107/S1600536810006860

**Published:** 2010-02-27

**Authors:** Vahid Amani, Nasser Safari, Hamid Reza Khavasi

**Affiliations:** a Department of Chemistry, Shahid Beheshti University, G. C., Evin, Tehran 1983963113, Iran

## Abstract

In the title mol­ecular salt, (C_11_H_10_N)[AuCl_4_], the Au^III^ atom adopts an almost regular square-planar coordination geometry and the dihedral angle between the aromatic rings of the 3-phenyl­pyridinium cation is 23.1 (3)°. In the crystal, the ions inter­act by way of N—H⋯Cl and C—H⋯Cl hydrogen bonds.

## Related literature

For related structures, see: Calleja *et al.* (2001 ▶); Faza­eli *et al.* (2010 ▶); Hasan *et al.* (1999 ▶); Hojjat Kashani *et al.* (2008 ▶); Johnson & Steed (1998 ▶); Safari *et al.* (2009 ▶); Yap *et al.* (1995 ▶); Yıldırım, Akkurt, Safari, Abedi *et al.* (2009 ▶); Yıldırım, Akkurt, Safari, Amani & McKee (2009 ▶); Zhang *et al.* (2006 ▶).
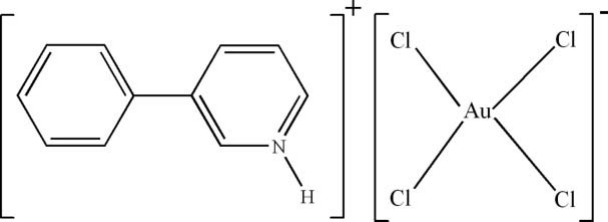


## Experimental

### 

#### Crystal data

(C_11_H_10_N)[AuCl_4_]
*M* *_r_* = 494.97
Triclinic, 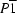

*a* = 7.7629 (9) Å
*b* = 8.5901 (11) Å
*c* = 11.0530 (15) Å
α = 94.106 (11)°
β = 107.125 (10)°
γ = 97.216 (10)°
*V* = 694.20 (15) Å^3^
*Z* = 2
Mo *K*α radiation
μ = 11.34 mm^−1^
*T* = 298 K
0.40 × 0.35 × 0.28 mm

#### Data collection

Stoe IPDSII diffractometer
Absorption correction: numerical (*X-RED*; Stoe & Cie, 2005 ▶) *T*_min_ = 0.067, *T*_max_ = 0.180
7980 measured reflections
3688 independent reflections
3513 reflections with *I* > 2σ(*I*)
*R*_int_ = 0.051

#### Refinement

*R*[*F* ^2^ > 2σ(*F* ^2^)] = 0.030
*wR*(*F* ^2^) = 0.083
*S* = 1.15
3688 reflections
154 parameters
H-atom parameters constrained
Δρ_max_ = 1.19 e Å^−3^
Δρ_min_ = −1.85 e Å^−3^

### 

Data collection: *X-AREA* (Stoe & Cie, 2005 ▶); cell refinement: *X-AREA*; data reduction: *X-AREA*; program(s) used to solve structure: *SHELXS97* (Sheldrick, 2008 ▶); program(s) used to refine structure: *SHELXL97* (Sheldrick, 2008 ▶); molecular graphics: *ORTEP-3* (Farrugia, 1997 ▶); software used to prepare material for publication: *WinGX* (Farrugia, 1999 ▶).

## Supplementary Material

Crystal structure: contains datablocks I, global. DOI: 10.1107/S1600536810006860/hb5341sup1.cif

Structure factors: contains datablocks I. DOI: 10.1107/S1600536810006860/hb5341Isup2.hkl

Additional supplementary materials:  crystallographic information; 3D view; checkCIF report

## Figures and Tables

**Table 1:** Selected bond lengths (Å)

Au1—Cl2	2.2740 (13)
Au1—Cl3	2.2754 (12)
Au1—Cl1	2.2762 (12)
Au1—Cl4	2.2766 (13)

**Table 2:** Hydrogen-bond geometry (Å, °)

*D*—H⋯*A*	*D*—H	H⋯*A*	*D*⋯*A*	*D*—H⋯*A*
N1—H1*A*⋯Cl3^i^	0.86	2.63	3.359 (7)	143
C1—H1⋯Cl4^i^	0.93	2.83	3.755 (7)	175

Symmetry code: (i) 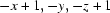.

**Fig. 1.**
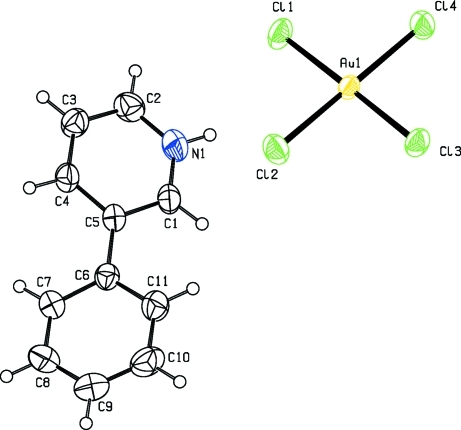
The molecular structure of (I). Displacement ellipsoids are drawn at the 40% probability level.

**Fig. 2.**
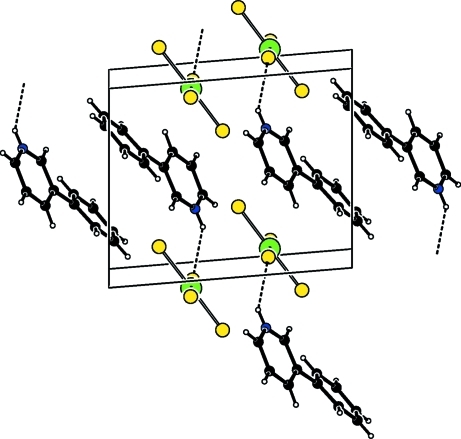
Unit-cell packing diagram for (I). Hydrogen bonds are shown as dashed lines.

## supplementary crystallographic information

### Comment

There are several proton transfer systems using HAuCl_4_ with proton acceptor molecules, such as [EMI][AuCl_4_], (II) and [BMI]_2_[AuCl_4_].2H_2_O, (III), (Hasan *et al.*, 1999), [H_2_bipy][AuCl_4_][Cl], (IV), (Zhang *et al.*, 2006), [H_7_O_3_][15-crown-5][AuCl_4_], (V) and [H_5_O_2_][benzo-15-crown-5]_2_[AuCl_4_], (VI), (Johnson & Steed, 1998), [H_5_O_2_]_2_[12-crown-4]_2_[AuCl_4_]_2_, (VII), [H_3_O][18-crown-6][AuCl_4_], (VIII) and [H_3_O][4-nitrobenzo-18-crown-6][AuCl_4_], (IX), (Calleja *et al.*, 2001), [DPpy.H][AuCl_4_], (X), (Yap *et al.*, 1995),[H_2_DA18C6][AuCl_4_].2H2O, (XI), (Hojjat Kashani *et al.*, 2008), [dafonium][dafone][AuCl_4_], (XII), (Safari *et al.*, 2009), [pz(py)_2_.H][AuCl_4_], (XIII), (Yıldırım, Akkurt, Safari, Amani & McKee 2009), [Ph_2_Phen.H][AuCl_4_], (XIV), (Yıldırım, Akkurt, Safari, Abedi *et al.*, 2009) and [TBA]_2_[AuCl_4_][Cl], (XV), (Fazaeli *et al.*, 2010) [Where EMI is 1-ethyl-3-methylimidazolium, BMI is 1-butyl-3-methylimidazolium, H_2_bipy is 2,2'-bipyridinium, DPpy.H is 2,6-diphenylpyridinium, H_2_DA18C6 is 1,10-diazonia-18-crown-6, dafonium is 9-oxo-4,5-diazafluoren-4-ium, dafone is 4,5-diazafluoren-9-one, pz(py)_2_.H is 2-(3-pyridin-2-ylpyrazin-2-yl)pyridinium, Ph_2_Phen.H is 2,9-dimethyl-4,7-diphenyl-1,10-phenanthrolin-1-ium and TBA is tribenzylammonium] have been synthesized and characterized by single-crystal X-ray diffraction methods. We report herein the synthesis and crystal structure of the title compound, (I).

The molecule of the title compound, (I), (Fig. 1), contains one independent protonated 3-phenylpyridinium cation and one [AuCl_4_]^-^ anion. The Au^III^ atom has a squareplanar environment defined by four Cl atoms. In [AuCl_4_]^-^ anion, the Au—Cl bond lengths and angles (Table 1) are within normal range (II, III, VII, VIII and IX).

In the crystal structure, intermolecular N—H···Cl and C—H···Cl hydrogen bonds (Table 2) link the molecules (Fig. 2), in which they may be effective in the stabilization of the structure.

### Experimental

A solution of 3-phenylpyridine (0.11 g, 0.09 ml, 0.74 mmol) in methanol (5 ml) was added to a solution of HAuCl_4_.3H_2_O, (0.29 g, 0.74 mmol) in acetonitrile (15 ml) and the resulting yellow solution was stirred for 30 min at 313 K. Then, it was left to evaporate slowly at room temperature. After five days, yellow blocks of (I) were isolated (yield 0.26 g; 71.0%).

### Refinement

All H atoms were positioned geometrically, with C—H=0.93Å for aromatics H and constrained to ride on their parent atoms, with U_iso_(H)=1.2U_eq_.

### Crystal data

**Table:**

(C_11_H_10_N)[AuCl_4_]	*Z* = 2
*M**_r_* = 494.97	*F*(000) = 460
Triclinic, *P*1	*D*_x_ = 2.368 Mg m^−^^3^
Hall symbol: -P 1	Mo *K*α radiation, λ = 0.71073 Å
*a* = 7.7629 (9) Å	Cell parameters from 984 reflections
*b* = 8.5901 (11) Å	θ = 1.9–29.2°
*c* = 11.0530 (15) Å	µ = 11.34 mm^−^^1^
α = 94.106 (11)°	*T* = 298 K
β = 107.125 (10)°	Block, yellow
γ = 97.216 (10)°	0.40 × 0.35 × 0.28 mm
*V* = 694.20 (15) Å^3^	

### Data collection

**Table:**

Stoe IPDS II diffractometer	3688 independent reflections
Radiation source: fine-focus sealed tube	3513 reflections with *I* > 2σ(*I*)
graphite	*R*_int_ = 0.051
Detector resolution: 0.15 mm pixels mm^-1^	θ_max_ = 29.2°, θ_min_ = 1.9°
rotation method scans	*h* = −10→10
Absorption correction: numerical (*X-RED*; Stoe & Cie, 2005)	*k* = −11→11
*T*_min_ = 0.067, *T*_max_ = 0.180	*l* = −15→15
7980 measured reflections	

### Refinement

**Table:**

Refinement on *F*^2^	Primary atom site location: structure-invariant direct methods
Least-squares matrix: full	Secondary atom site location: difference Fourier map
*R*[*F*^2^ > 2σ(*F*^2^)] = 0.030	Hydrogen site location: inferred from neighbouring sites
*wR*(*F*^2^) = 0.083	H-atom parameters constrained
*S* = 1.15	*w* = 1/[σ^2^(*F*_o_^2^) + (0.049*P*)^2^ + 0.4225*P*] where *P* = (*F*_o_^2^ + 2*F*_c_^2^)/3
3688 reflections	(Δ/σ)_max_ = 0.001
154 parameters	Δρ_max_ = 1.19 e Å^−^^3^
0 restraints	Δρ_min_ = −1.85 e Å^−^^3^

### Special details

**Table:**

Geometry. All esds (except the esd in the dihedral angle between two l.s. planes) are estimated using the full covariance matrix. The cell esds are taken into account individually in the estimation of esds in distances, angles and torsion angles; correlations between esds in cell parameters are only used when they are defined by crystal symmetry. An approximate (isotropic) treatment of cell esds is used for estimating esds involving l.s. planes.
Refinement. Refinement of *F*^2^ against ALL reflections. The weighted *R*-factor wR and goodness of fit *S* are based on *F*^2^, conventional *R*-factors *R* are based on *F*, with *F* set to zero for negative *F*^2^. The threshold expression of *F*^2^ > σ(*F*^2^) is used only for calculating *R*-factors(gt) etc. and is not relevant to the choice of reflections for refinement. *R*-factors based on *F*^2^ are statistically about twice as large as those based on *F*, and *R*- factors based on ALL data will be even larger.

### Fractional atomic coordinates and isotropic or equivalent isotropic displacement parameters (Å^2^)

**Table:**

	*x*	*y*	*z*	*U*_iso_*/*U*_eq_	
C1	0.3073 (8)	0.3645 (7)	0.7222 (6)	0.0534 (13)	
H1	0.4141	0.3203	0.7415	0.064*	
C2	−0.0102 (9)	0.3328 (8)	0.6148 (7)	0.0593 (14)	
H2	−0.1157	0.2683	0.5638	0.071*	
C3	−0.0149 (8)	0.4824 (7)	0.6606 (6)	0.0556 (13)	
H3	−0.1242	0.5229	0.6395	0.067*	
C4	0.1433 (8)	0.5748 (6)	0.7388 (6)	0.0487 (11)	
H4	0.1386	0.6775	0.7691	0.058*	
C5	0.3102 (7)	0.5176 (6)	0.7733 (5)	0.0412 (9)	
C6	0.4797 (7)	0.6118 (6)	0.8579 (5)	0.0429 (9)	
C7	0.4751 (9)	0.7355 (7)	0.9442 (6)	0.0556 (13)	
H7	0.3628	0.7566	0.9497	0.067*	
C8	0.6310 (11)	0.8273 (9)	1.0215 (7)	0.0693 (18)	
H8	0.6239	0.9105	1.0776	0.083*	
C9	0.7981 (10)	0.7962 (10)	1.0160 (7)	0.0676 (17)	
H9	0.9042	0.8600	1.0669	0.081*	
C10	0.8084 (9)	0.6706 (10)	0.9353 (8)	0.0721 (19)	
H10	0.9217	0.6470	0.9342	0.087*	
C11	0.6485 (8)	0.5781 (8)	0.8545 (6)	0.0591 (14)	
H11	0.6556	0.4944	0.7989	0.071*	
N1	0.1525 (9)	0.2805 (7)	0.6455 (6)	0.0651 (14)	
H1A	0.1565	0.1871	0.6136	0.078*	
Au1	0.377224 (19)	0.071414 (17)	0.338315 (15)	0.03590 (7)	
Cl1	0.08562 (18)	0.08586 (18)	0.33626 (17)	0.0559 (3)	
Cl2	0.4746 (2)	0.30376 (17)	0.46750 (16)	0.0571 (3)	
Cl3	0.67028 (17)	0.05728 (17)	0.34422 (16)	0.0509 (3)	
Cl4	0.28061 (19)	−0.15918 (17)	0.20634 (17)	0.0581 (3)	

### Atomic displacement parameters (Å^2^)

**Table:**

	*U*^11^	*U*^22^	*U*^33^	*U*^12^	*U*^13^	*U*^23^
C1	0.051 (3)	0.046 (3)	0.063 (3)	0.013 (2)	0.019 (2)	−0.006 (2)
C2	0.056 (3)	0.052 (3)	0.064 (3)	−0.004 (2)	0.016 (3)	−0.002 (3)
C3	0.046 (3)	0.052 (3)	0.067 (3)	0.008 (2)	0.016 (2)	0.004 (3)
C4	0.053 (3)	0.039 (2)	0.057 (3)	0.012 (2)	0.019 (2)	0.003 (2)
C5	0.047 (2)	0.037 (2)	0.045 (2)	0.0093 (18)	0.0195 (19)	0.0048 (17)
C6	0.046 (2)	0.042 (2)	0.044 (2)	0.0103 (18)	0.0162 (19)	0.0077 (18)
C7	0.057 (3)	0.054 (3)	0.053 (3)	0.013 (2)	0.014 (2)	−0.005 (2)
C8	0.072 (4)	0.066 (4)	0.057 (3)	0.009 (3)	0.004 (3)	−0.010 (3)
C9	0.053 (3)	0.078 (4)	0.064 (4)	0.005 (3)	0.007 (3)	0.011 (3)
C10	0.042 (3)	0.098 (5)	0.075 (4)	0.012 (3)	0.016 (3)	0.011 (4)
C11	0.050 (3)	0.066 (3)	0.064 (3)	0.012 (3)	0.023 (3)	−0.001 (3)
N1	0.071 (3)	0.045 (2)	0.074 (3)	0.011 (2)	0.018 (3)	−0.011 (2)
Au1	0.02898 (10)	0.03416 (10)	0.04446 (11)	0.00715 (6)	0.01061 (7)	0.00261 (7)
Cl1	0.0343 (5)	0.0527 (6)	0.0830 (9)	0.0122 (5)	0.0208 (6)	0.0012 (6)
Cl2	0.0560 (7)	0.0460 (6)	0.0653 (8)	0.0017 (5)	0.0195 (6)	−0.0122 (6)
Cl3	0.0307 (5)	0.0498 (6)	0.0711 (8)	0.0074 (4)	0.0153 (5)	−0.0007 (6)
Cl4	0.0428 (6)	0.0492 (6)	0.0737 (9)	0.0055 (5)	0.0108 (6)	−0.0164 (6)

### Geometric parameters (Å, °)

**Table:**

C1—N1	1.333 (8)	C7—H7	0.9300
C1—C5	1.389 (7)	C8—C9	1.375 (12)
C1—H1	0.9300	C8—H8	0.9300
C2—N1	1.351 (9)	C9—C10	1.376 (11)
C2—C3	1.356 (9)	C9—H9	0.9300
C2—H2	0.9300	C10—C11	1.403 (9)
C3—C4	1.384 (8)	C10—H10	0.9300
C3—H3	0.9300	C11—H11	0.9300
C4—C5	1.400 (7)	N1—H1A	0.8600
C4—H4	0.9300	Au1—Cl2	2.2740 (13)
C5—C6	1.470 (8)	Au1—Cl3	2.2754 (12)
C6—C11	1.388 (8)	Au1—Cl1	2.2762 (12)
C6—C7	1.388 (7)	Au1—Cl4	2.2766 (13)
C7—C8	1.368 (9)		
			
N1—C1—C5	120.5 (5)	C7—C8—C9	119.8 (7)
N1—C1—H1	119.7	C7—C8—H8	120.1
C5—C1—H1	119.7	C9—C8—H8	120.1
N1—C2—C3	117.9 (6)	C8—C9—C10	120.0 (7)
N1—C2—H2	121.0	C8—C9—H9	120.0
C3—C2—H2	121.0	C10—C9—H9	120.0
C2—C3—C4	119.9 (6)	C9—C10—C11	120.2 (6)
C2—C3—H3	120.0	C9—C10—H10	119.9
C4—C3—H3	120.0	C11—C10—H10	119.9
C3—C4—C5	121.7 (5)	C6—C11—C10	119.8 (6)
C3—C4—H4	119.2	C6—C11—H11	120.1
C5—C4—H4	119.2	C10—C11—H11	120.1
C1—C5—C4	115.8 (5)	C1—N1—C2	124.0 (5)
C1—C5—C6	121.2 (5)	C1—N1—H1A	118.0
C4—C5—C6	123.0 (4)	C2—N1—H1A	118.0
C11—C6—C7	118.3 (5)	Cl2—Au1—Cl3	89.51 (5)
C11—C6—C5	120.8 (5)	Cl2—Au1—Cl1	89.92 (6)
C7—C6—C5	120.8 (5)	Cl3—Au1—Cl1	178.98 (5)
C8—C7—C6	121.8 (6)	Cl2—Au1—Cl4	179.07 (6)
C8—C7—H7	119.1	Cl3—Au1—Cl4	90.20 (5)
C6—C7—H7	119.1	Cl1—Au1—Cl4	90.38 (5)
			
N1—C2—C3—C4	−1.5 (10)	C11—C6—C7—C8	2.5 (10)
C2—C3—C4—C5	−0.5 (10)	C5—C6—C7—C8	−178.3 (6)
N1—C1—C5—C4	−0.2 (9)	C6—C7—C8—C9	−1.0 (12)
N1—C1—C5—C6	−180.0 (6)	C7—C8—C9—C10	−1.6 (13)
C3—C4—C5—C1	1.3 (9)	C8—C9—C10—C11	2.7 (12)
C3—C4—C5—C6	−178.9 (5)	C7—C6—C11—C10	−1.4 (10)
C1—C5—C6—C11	23.1 (8)	C5—C6—C11—C10	179.4 (6)
C4—C5—C6—C11	−156.7 (6)	C9—C10—C11—C6	−1.1 (12)
C1—C5—C6—C7	−156.1 (6)	C5—C1—N1—C2	−1.9 (11)
C4—C5—C6—C7	24.1 (8)	C3—C2—N1—C1	2.8 (11)

### Hydrogen-bond geometry (Å, °)

**Table:**

*D*—H···*A*	*D*—H	H···*A*	*D*···*A*	*D*—H···*A*
N1—H1A···Cl3^i^	0.86	2.63	3.359 (7)	143
C1—H1···Cl4^i^	0.93	2.83	3.755 (7)	175

Symmetry codes: (i) −*x*+1, −*y*, −*z*+1.
